# Changes in cardiac troponins during hemodialysis depend on hemodialysis membrane and modality: a randomized crossover trial

**DOI:** 10.1093/ckj/sfad297

**Published:** 2023-12-05

**Authors:** Michael Kolland, Jascha Amenitsch, Nikolaus Schreiber, Noemi Ginthör, Max Schuller, Regina Riedl, Peter P Rainer, Daniel Schneditz, Tobias Niedrist, Kathrin Eller, Benedikt Krietemeyer, Alexander R Rosenkranz, Alexander H Kirsch

**Affiliations:** Department of Internal Medicine, Division of Nephrology, Medical University of Graz, Graz, Austria; Department of Internal Medicine, Division of Nephrology, Medical University of Graz, Graz, Austria; Department of Internal Medicine, Division of Nephrology, Medical University of Graz, Graz, Austria; Department of Internal Medicine, Division of Nephrology, Medical University of Graz, Graz, Austria; Department of Internal Medicine, Division of Nephrology, Medical University of Graz, Graz, Austria; Institute for Medical Informatics, Statistics and Documentation, Medical University of Graz, Graz, Austria; Department of Internal Medicine, Division of Cardiology, Medical University of Graz, BioTechMed Graz, Graz, Austria; Division of Physiology, Otto Loewi Research Center, Medical University of Graz, Graz, Austria; Clinical Institute of Medical and Chemical Laboratory Diagnostics, Medical University of Graz, Graz, Austria; Department of Internal Medicine, Division of Nephrology, Medical University of Graz, Graz, Austria; Department of Internal Medicine, Division of Nephrology, Medical University of Graz, Graz, Austria; Department of Internal Medicine, Division of Nephrology, Medical University of Graz, Graz, Austria; Department of Internal Medicine, Division of Nephrology, Medical University of Graz, Graz, Austria

To the Editor,

The diagnosis of acute coronary syndromes (ACS) is challenging in hemodialysis (HD) patients. Chronic HD patients with acute myocardial infarction (AMI) are less likely to present with chest pain (44.4% versus 68.3%) or show ST elevation (19.1% versus 35.9%) [1] but often show unspecific symptoms, such as hypotension (28.7%) or nausea/vomiting (11.7%), especially during dialysis treatments [2].

Thus, diagnosis of AMI may frequently be delayed or missed due to atypical presentation and relies heavily on biomarkers [i.e. cardiac troponins (cTn)]. Reports on cardiac troponin T (cTnT) kinetics during HD are heterogeneous [3]. Likewise, reports on cardiac troponin I (cTnI) are inconsistent [4, 5] and baseline levels in HD patients are commonly above established recommended thresholds. Importantly, dialysis membranes and modalities differ significantly in permeability for larger molecules: “low-flux” membranes with permeability for molecules with a molecular mass of up to 5 kDa, “high-flux” up to 20 kDa and the newest, “medium cut-off” (MCO) membranes, up to 45 kDa. With molecular masses of approximately 39 kDa and 26 kDa for cTnT and cTnI, respectively, clearances differ depending on the type of membrane and amount of filtration used in dialysis.

Currently, neither the European Society of Cardiology (ESC) [6] nor American Heart Association (AHA) [7] guidelines specify the diagnostic algorithm and use of biomarkers for patients during HD. A consensus report from the SONG-HD MI Expert Working group concluded there to be insufficient evidence for standard dialysis to impact the diagnosis of AMI [8]. In this study, we aimed to explore intradialytic cTn changes with routinely used HD membranes and treatment modalities to aid in the interpretation of cTn levels in dialysis patients.

Detailed methods are provided in the Supplementary data. In brief, in this randomized, controlled crossover study, asymptomatic, clinically stable patients at least 18 years of age on chronic HD were randomized to a sequence of one treatment session with low-flux HD, high-flux HD, hemodiafiltration (HDF) and MCO HD. Cardiac troponins were measured before dialysis, after 1 h and immediately after HD to examine changes of cardiac troponins.

Twenty patients were randomized, but one patient was excluded from analysis due to non-ST elevation ACS during dialysis with substantial distortion on the absolute values of the results, thus a per protocol analysis including 19 patients (47.4% female) with a mean age of 65.5 ± 13.4 years and a median of 19 months (min. 3, max. 165) on dialysis was conducted. Sixty-eight percent had a history of coronary artery disease (CAD) and 36.8% had previously suffered an AMI. Baseline characteristics are provided in Supplementary data, Table S1.

In mixed model analysis for the relative difference in cTnT, no significant sequence or period effects were observed for relative changes from baseline to 1 h (sequence: *P* = .45, period: *P* = .97).

Kinetics of cTn are shown in Fig. 1 and Table 1. The effect of membrane on the relative change on cTnT differed significantly (*P* < .001). Significantly different relative changes after 1 h were observed for MCO [least square mean (LSM) −21.9; 95% confidence interval (CI) −27.3 to −16.6%] compared with low-flux (LSM +2.2; 95% CI −3.2 to 7.5%, *P* < .001) and MCO to high-flux (LSM −6.8; 95% CI −12.2 to −1.5%, *P* < .001). No difference was observed for MCO versus HDF treatment with high-flux membrane (LSM −21.2; 95% CI −26.6 to −15.7%, *P* = .81). Similar results were observed post-HD.

**Figure 1:**
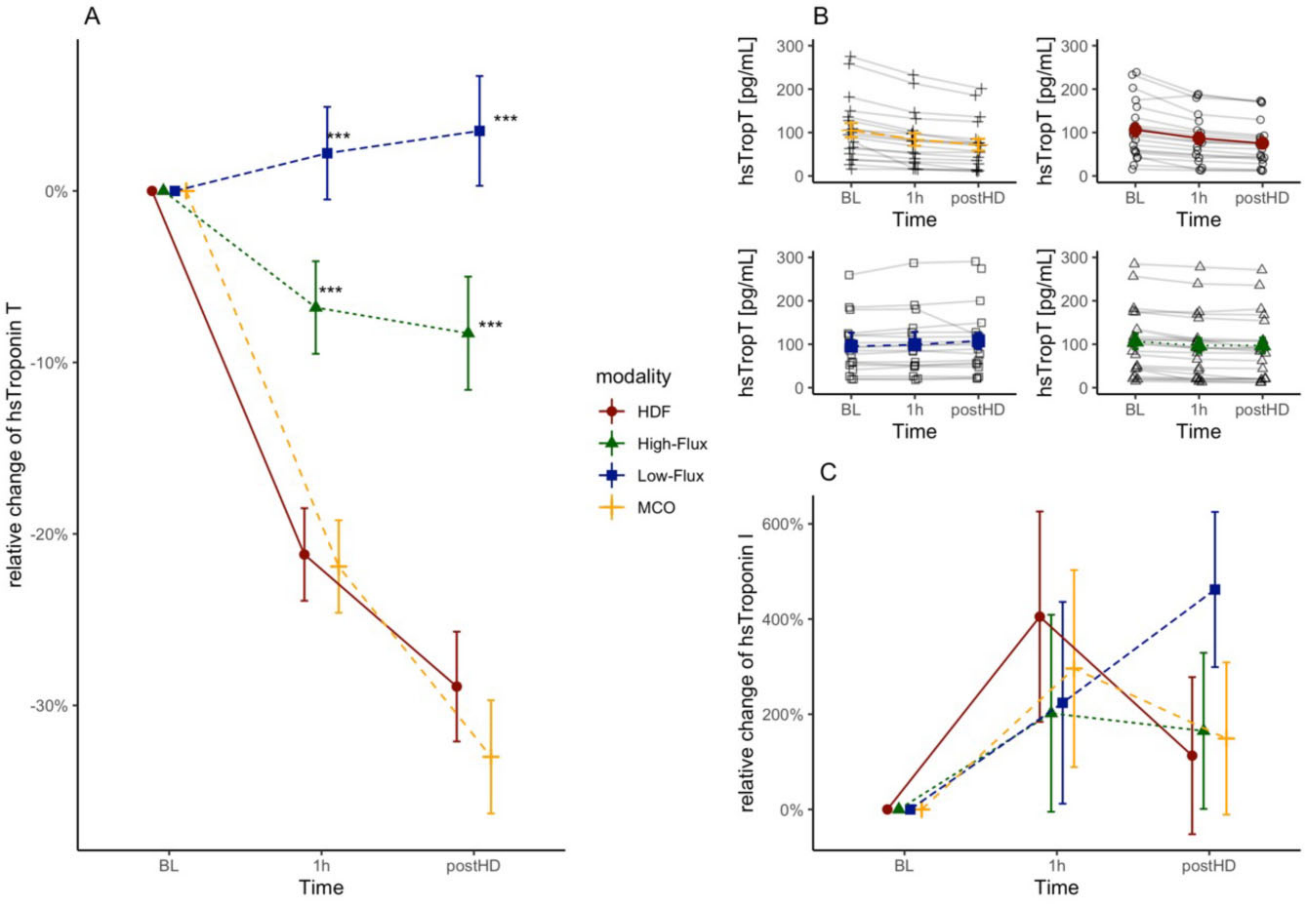
(**A**) Relative changes (presented as LSM ± SE) of cTnT according to treatment modality. (**B**) Absolute changes in pg/mL of cTnT according to treatment modality after 1 h of treatment and post-HD. (**C**) Relative changes of cTnI (presented as LSM ± SE) according to different treatment modalities. BL, baseline; SE, standard error. ***P < .001 refer to differences to MCO cTn levels according to treatment modalities.

**Table 1:** Troponin kinetics according to membrane characteristics and treatment modality.

			High-flux HD		Low-flux HD		MCO		HDF	
Troponin T	Baseline (pg/mL), mean (± SD)	0 h	101.3 (± 74.4)		107.7 (± 82.1)		105.4 (± 71.7)		103.2 (± 67.1)	
	Absolute differences (pg/mL), LSM (95% CI)	1 h	−6.4 (−12.8 to 0.0)	*P* = .049	2.3 (−4.1 to 8.6)	*P* = .48	−21.2 (−27.6 to −14.8)	*P* < .001	−20.2 (−26.8 to −13.7)^a^	*P* < .001
		Post-HD	−7.7 (−17.2 to 1.8)^a^	*P* = .11	0.3 (−9.0 to 9.6)	*P* = .96	−31.4 (−40.9 to −21.9)	*P* < .001	−27.8 (−37.1 to −18.5)^a^	*P* < .001
	Relative difference (%), LSM (95% CI)	1 h	−6.8 (−12.2 to −1.5)	*P* = .013	2.2 (−3.2 to 7.5)	*P* = .42	−21.9 (−27.3 to −16.6)	*P* < .001	−21.2 (−26.6 to −15.7)	*P* < .001
		Post-HD	−8.3 (−15.0 to −1.7)^a^	*P* = .015	3.5 (−3.0 to 9.9)	*P* = .28	−33.0 (−39.6 to −26.4)	*P* < .001	−28.9 (−35.3 to −22.4)	*P* < .001
Troponin I	Baseline (pg/mL), mean (± SD)	0 h	112.4 (± 159.3)		78.1 (± 87.2)^a^		73.8 (± 89.9)		89.3 (±145.5)^a^	
	Absolute differences (pg/mL), LSM (95% CI)	1 h	−10.3 (−81.5 to 60.8)	*P* = .77	40.5 (−32.2 to 113.2)^a^	*P* = .27	53.6 (−17.5 to 124.7)	*P* = .14	48.8 (−26.9 to 124.6)^a^	*P* = .20
		Post-HD	20.9 (−66.4 to 108.0)^a^	*P* = .63	102.1 (15.4 to 188.8)^a^	*P* = .022	47.7 (−37.1 to 132.4)	*P* = .26	−1.0 (−88.8 to 86.8)^a^	*P* = .98
	Relative difference (%), LSM (95% CI)	1 h	201.8 (−215.4 to 618.9)	*P* = .34	223.6 (−202.9 to 650.1)^a^	*P* = .30	295.7 (−121.3 to 712.6)	*P* = .16	405.2 (−38.9 to 849.3)^a^	*P* = .07
		Post-HD	165.3 (−164.6 to 495.2)^a^	*P* = .32	462.0 (133.8 to 790.1)^a^	*P* = .007	149.1 (−171.7 to 469.9)	*P* = .35	112.7 (−219.6 to 445.0)	*P* = .50
Troponin T corrected for hemo-concentration	Absolute differences (pg/mL), LSM (95% CI)	1 h	−16.8 (−26.7 to −6.9)	*P* = .001	−3.4 (−13.4 to 6.6)	*P* = .40	−26.4 (−36.1 to −16.7)	*P* < .0001	−25.2 (−35.8 to −14.6)^a^	*P* < .0001
		Post-HD	−23.1 (−37.0 to −9.2)^a^	*P* = .002	−10.1 (−24.0 to 3.9)	*P* = .15	−40.9 (−54.6 to −27.4)	*P* < .0001	−35.7 (−49.5 to −21.9)^a^	*P* < .0001
	Relative difference (%), LSM (95% CI)	1 h	−13.9 (−19.7 to −8.2)	*P* < .0001	−2.9 (−8.7 to 2.9)	*P* = .32	−27.1 (−32.7 to −21.5)	*P* < .0001	−26.3 (−32.6 to −20.0)	*P* < .0001
		Post-HD	−21.4 (−28.5 to −14.2)^a^	*P* < .0001	−8.4 (−15.5 to −1.2)	*P* = .02	−41.9 (−48.8 to −35.0)	*P* < .0001	−36.9 (−43.9 to −29.8)	*P* < .0001
Troponin I corrected for hemoconcentration	Absolute differences (pg/mL), LSM (95% CI)	1 h	−25.5 (−88.6 to 37.6)	*P* = .42	35.5 (−29.9 to 100.8)^a^	*P* = .28	45.6 (−16.1 to 107.3)	*P* = .14	18.4 (−53.8 to 90.5)^a^	*P* = .61
		Post-HD	6.1 (−74.5 to 86.8)^a^	*P* = .88	83.1 (0.0 to 166.2)^a^	*P* = .05	28.1 (−47.6 to 103.8)	*P* = .46	8.5 (−74.0 to 91.0)^a^	*P* = .84
	Relative difference (%), LSM (95% CI)	1 h	208.1 (−86.8 to 503.0)	*P* = .16	219.4 (−86.0 to 524.8)^a^	*P* = .15	268.3 (−20 to 556.6)	*P* = .06	69.6 (−267.7 to 406.9)^a^	*P* = .67
		Post-HD	146.80 (−170.8 to 464.4)^a^	*P* = .36	450.2 (123.2 to 777.2)^a^	*P* = .008	98.32 (−199.6 to 396.3)	*P* = .51	110.44 (−214.2 to 435.1)	*P* = .49

LSMs derived by linear mixed model analysis.

^a^ *n* = 17–18 due to missing data.

Comparisons and *P*-values refer to differences relative to baselines.

For absolute changes, LSM for MCO were −21.2 (95% CI −27.6 to −14.8 pg/mL), −6.4 (95% CI −12.8 to −0.0 pg/mL) for high-flux, −20.2 (95% CI −26.8 to −13.7 pg/mL) for HDF treatment and +2.3 (95% CI −4.1 to 8.6 pg/mL) for low-flux hemodialysis after 1 h. There were no significant effects of sequence, period or membrane effects observed for relative changes of cTnI from baseline to 1 h (sequence: *P* = .14, period: *P* = .68, membrane: *P* = .91) and from baseline to post-HD (sequence: *P* = .58, period = 0.23, membrane = 0.41).

The results were concordant but more pronounced when accounting for ultrafiltration-induced hemoconcentration, with even higher observed relative and absolute changes of cTnT for high-flux HD, HDF and MCO HD, while the increase of cTnT seen in low-flux HD vanished. After correction for hemoconcentration, there was a statistically significant increase of cTnI seen in low-flux HD (Table 1).

In this randomized crossover trial we found significant decreases of cTnT with MCO and HDF, and smaller decreases with high-flux HD but not with low-flux HD in clinically stable patients. These data support that cTnT is cleared significantly by high-flux and MCO HD, as well as by HDF. The increase seen in low-flux is primarily due to hemoconcentration in the setting of absent cTnT clearance, and disappeared when correcting for hemoconcentration using pre/post-hematocrit [9]. If hemoglobin concentrations, hematocrit or the relative blood volumes are available at measuring times, the increase can be predicted. The effect of hemoconcentration is also present in other treatment modes and one can discuss that true intradialytic reductions are probably even larger than derived from uncorrected concentrations (Table 1). However, while hemoconcentration artificially raises cTnT levels in low-flux HD, in the clinical setting of suspected ongoing myocardial ischemia, adjusting for hemoconcentration will not be feasible and there are no data on whether this will alter test characteristics. A measurement 1 h after dialysis would show the equilibrated concentrations and reveal effects of hemoconcentration as well. Increases of cTn concentrations post-HD, indicating equilibration, have been described previously [10].

A possible reason for the reduction of cTnT being more pronounced with MCO compared with HDF is that, first, at low blood flow rates, as observed in our study, MCO seems to be more effective than HDF in terms of larger middle molecule clearances [11], and second, because total convective volume was rather low in our study.

There was no clear trend in cTnI kinetics and values varied substantially, which does not support clinical use of cTnI in this setting. This is surprising and requires further clarification, as cTnI is smaller than cTnT and a larger drop would therefore be expected with cTnT in high-flux treatments. Insignificant clearance of cTnI could also be explained by a high net negative charge, possibly caused by high phosphorylation [12], by protein binding and ultrafiltration-induced hemoconcentration, or membrane adsorption. After a cardiac insult (non-ST elevation myocardial infarction), cTnI increases faster than cTnT in a regular population [13]. Since myocardial stunning may lead to a rise of cTn, and we did not assess the occurrence of stunning, one might assume that there might be a distinct difference in release of cTnI and cTnT, with extrarenal clearance [14], which may contribute to the large intraindividual changes during each session. Given the multiple reasons for cTnT elevation in HD patients, a more specific biomarker for myocardial ischemia is highly desirable in this patient population.

Considering the algorithms of the 2023 ESC Guidelines [6], a 1 h delta of cTnT >5 pg/mL would be missed in most patients. Furthermore, a delta of >20% of cTn, as recommended by the standardised outcomes in nephrology group–haemodialysis (SONG-HD) Expert Working group [8], occurs without evidence of ACS when MCO membranes or HDF are applied. Any uncorrected increase in cTnT during dialysis (except low-flux HD) would be sensitive but not specific for AMI. While higher baseline cTnT levels in renal patients are well-described, the influence of HD on cTnT may frequently not be considered and should be included when consulting cardiology for a patient evaluation for ACS.

The main limitation is the patient population, a sizable proportion of whom had preexisting CAD, and which deliberately included exclusively stable HD patients, without suspected ongoing myocardial ischemia, to study the effect of dialysis without having to account for differences in the myocardial release of troponins. Thus, the generalizability for other patient populations is limited, specifically for patients with ACS.

Taken together, current diagnostic algorithms cannot be uncritically applied to assess AMI during hemodialysis sessions and troponin kinetics need to be interpreted cautiously in concert with clinical, electrocardiogram and imaging parameters in patients undergoing HD.

## Supplementary Material

sfad297_Supplemental_File Patients’ baseline characteristics and methods are provided as part of the Supplementary data.

## Data Availability

Data available upon reasonable request to the corresponding author.
