# Supplementary material for: Changes in cardiac troponins during hemodialysis depend on hemodialysis membrane and modality: a randomized crossover trial
Source: Clin Kidney J. 2023 Dec 5;17(1):sfad297. doi: 10.1093/ckj/sfad297 (PMC10783248; doi:10.1093/ckj/sfad297)
Supplement: sfad297_Supplemental_File — Patients’ baseline characteristics and methods are provided as part of the Supplementary data. [file sfad297_supplemental_file.docx]

**Supplementary Materials**

**Supplementary Table**

Table S1 Baseline characteristics

| **Characteristic** | **All patients** (n=19) |
| --- | --- |
| Gender (percent, %)  Male  Female | 10 (52.6%)  9 (47.4%) |
| Age (years) (mean±SD) | 65.5± 13.4 |
| Dialysis vintage (days) (median, min and max) | 19 months (min. 3, max. 165) |
| Comorbidities (percent,%)  Cardiovascular disease  Diabetes mellitus  Arterial Hypertension  Left ventricular hypertrophy  Previous myocardial infarction  Smoking  Non-smoker  Present  Former | 13 (68%)  6 (31.6%)  17 (89.5%)  14 (73.7%)  7 (36.8%)  12 (63.2%)  1 (5.4%)  6 (31.6%) |
| Underlying renal disease (percent, %)  Diabetic kidney disease  Hypertensive/Vascular  Glomerulonephritis  Cystic Kidney Disease  Other/Unknown | 3 (15.8%)  4 (21.0%)  2 (10.6%)  3 (15.8%)  7 (36.8%) |
| Type of access (percent, %)  Catheter  Fistula | 16 (84.2%)  3 (15.8%) |
| Residual renal function (percent, %)  ≥ 500mL/day  < 500mL/day | 6 (31.6%) 13 (68.4%) |

**Supplementary References**

1. Fitzgerald RL, Hollander JE, Peacock WF, Limkakeng AT, Breitenbeck N, Blechschmidt K, et al. Analytical performance evaluation of the Elecsys® Troponin T Gen 5 STAT assay. Clinica Chimica Acta. 2019;495:522-8.

2. Krintus M, Kozinski M, Boudry P, Capell NE, Koller U, Lackner K, et al. European multicenter analytical evaluation of the Abbott ARCHITECT STAT high sensitive troponin I immunoassay. Clin Chem Lab Med. 2014;52(11):1657-65.

3. Thygesen K, Alpert JS, Jaffe AS, Chaitman BR, Bax JJ, Morrow DA, et al. Fourth Universal Definition of Myocardial Infarction (2018). Circulation. 2018;138(20):e618-e51.

4. Kirsch AH, Lyko R, Nilsson LG, Beck W, Amdahl M, Lechner P, et al. Performance of hemodialysis with novel medium cut-off dialyzers. Nephrol Dial Transplant. 2017;32(1):165-72.

5. Schneditz D, Putz-Bankuti C, Ribitsch W, Schilcher G. Correction of plasma concentrations for effects of hemoconcentration or hemodilution. ASAIO J. 2012;58(2):160-2.

**Supplementary Methods**

*Study Subjects*

Patients at least 18 years of age on chronic HD for at least 3 months were eligible to participate. Pregnant female patients were excluded.

*Objectives*

The primary aim was to compare the relative and absolute changes of high sensitivity cTnT and cTnI from baseline to after 1 hour and directly after HD treatment for different hemodialysis treatments. Secondary outcomes included absolute and relative changes of cTnT and cTnI during and after treatment.

*Treatments*

After obtaining informed consent, patients were randomized to a treatment sequence (1:1:1:1 Williams design) with different modalities including low-flux HD, high-flux HD (FX10 Dialyzer and FxCor Diax 800 (both Fresenius Medical Care, Bad Homburg, Germany)), online post-dilution hemodiafiltration (HDF (FxCor Diax 800)), and MCO-HD (Theranova 400 (Baxter, Deerfield, Illinois, USA)), for 4 consecutive mid-week dialysis sessions in cross-over design. Therefore, each patient was treated with each membrane, respectively modality once and, vice versa, each modality or membrane was used once for all included patients. Dialysis treatments were standardized, with a dialysate temperature of 35.5°C, dialysate calcium 1.25mmol/L, bicarbonate of 30mmol/L and sodium, potassium and ultrafiltration rate according to the caretaking provider. Blood was drawn before, after 1 hour, and immediately after HD. Troponin diagnostics were performed using the Elecsys^®^ Troponin T-hs (Roche Diagnostics GmbH, Mannheim, Germany) (with a Coefficients of variation <10% at the 99th percentile upper reference limit (URL)(1) and Alinity I STAT High Sensitive Troponin-I Reagent Kit (Abbott Ireland Diagnostics Division, Lisnamuck, Longford, Ireland)(2), both meeting precision requirements according to the Fourth Universal Definition of Myocardial Infarction for high-sensitivity troponin assays)(3)

*Statistical Analysis*

Sample size calculation was based on a previous study on middle molecule clearance in a similar size range (4). In a publication by Kirsch et al. (2016), relative reduction in YKL-40 for MCO membrane, high-flux membrane and HDF treatment was investigated in 20 patients. For MCO a relative reduction (pre to post treatment session of about 4 hours) of 63.6% (2.2%), for high-flux: 29.8% (2.2%) and for HDF: 44.8% (2.2%), were observed.(4) The molecular weight of Troponin T is similar to the molecular weight of YKL-40 (hs-Troponin T: 39kDa and YKL-40: 40kDa). Therefore, similar results are expected (i.e., difference between MCO and HDF of about 20% after 4 hours). Since, in this study, the difference after one hour will be the primary focus, smaller differences between MCO and HDF are assumed (between 5%-10%) with a standard deviation of 5% (summarized below). Based on a sample size calculation with an alpha of 1.67%, a power of 90% and a two-sided paired t-test, 20 subjects need to be included. Assuming a drop out of 4 patients, we aimed to recruit 24 patients.

Primary and secondary endpoints were analyzed using linear mixed models with subject as random effect and sequence (1-4), period (1-4) and treatment (1-4) as fixed effects. Differences between the treatments (MCO vs. low-flux, high-flux and HDF) were presented as least square means (LSM) with 95% confidence intervals (CIs). A p-value of <0.0167 indicates statistical significance for the primary objective (Bonferroni correction), otherwise <0.05.

Endpoints were further corrected for ultrafiltration-induced hemoconcentration according to Schneditz et al.(5):

hp=H1/H0*(100-H0)/(100-H1),

Factor hp…hemoconcentration
H0…Hematocrit at baseline

H1… Hematocrit at sampling time points at 1 hour, respectively post HD.

The study was approved by the local IRB (34-306ex21/22) and the local competent authorities (BASG, reference number 101037133) and registered at clinicaltrials.gov (NCT05439681).
